# Using mid-infrared spectroscopy to increase GWAS power to detect QTL associated with blood urea nitrogen

**DOI:** 10.1186/s12711-022-00719-5

**Published:** 2022-04-18

**Authors:** Irene van den Berg, Phuong N. Ho, Tuan V. Nguyen, Mekonnen Haile-Mariam, Timothy D. W. Luke, Jennie E. Pryce

**Affiliations:** 1 AgriBio, Centre of AgriBioscience, Agriculture Victoria, 5 Ring Road, Bundoora, VIC 3082 Australia; 2 School of Applied Systems Biology, La Trobe University, Bundoora, VIC 3083 Australia

## Abstract

Blood urea nitrogen (BUN) is an indicator trait for urinary nitrogen excretion. Measuring BUN level requires a blood sample, which limits the number of records that can be obtained. Alternatively, BUN can be predicted using mid-infrared (MIR) spectroscopy of a milk sample and thus records become available on many more cows through routine milk recording processes. The genetic correlation between MIR predicted BUN (MBUN) and BUN is 0.90. Hence, genetically, BUN and MBUN can be considered as the same trait. The objective of our study was to perform genome-wide association studies (GWAS) for BUN and MBUN, compare these two GWAS and detect quantitative trait loci (QTL) for both traits, and compare the detected QTL with previously reported QTL for milk urea nitrogen (MUN). The dataset used for our analyses included 2098 and 18,120 phenotypes for BUN and MBUN, respectively, and imputed whole-genome sequence data. The GWAS for MBUN was carried out using either the full dataset, the 2098 cows with records for BUN, or 2000 randomly selected cows, so that the dataset size is comparable to that for BUN. The GWAS results for BUN and MBUN were very different, in spite of the strong genetic correlation between the two traits. We detected 12 QTL for MBUN, on bovine chromosomes 2, 3, 9, 11, 12, 14 and X, and one QTL for BUN on chromosome 13. The QTL detected on chromosomes 11, 14 and X overlapped with QTL detected for MUN. The GWAS results were highly sensitive to the subset of records used. Hence, caution is warranted when interpreting GWAS based on small datasets, such as for BUN. MBUN may provide an attractive alternative to perform a more powerful GWAS to detect QTL for BUN.

**Supplementary Information:**

The online version contains supplementary material available at 10.1186/s12711-022-00719-5.

## Background

The dairy industry is under increasing pressure to improve its sustainability and reduce its environmental footprint. Reducing urinary nitrogen excretion from grazing dairy cattle would lead to a reduced environmental impact [1]. While it would be difficult to collect a large enough dataset with urinary nitrogen excretion records to allow genetic selection, related indicator traits are more readily available. Blood urea nitrogen (BUN) is a biomarker for urinary nitrogen excretion [2]. However, direct measures of BUN level remain challenging to obtain because collecting blood samples routinely may not be feasible on most dairy farms. Alternatively, BUN can be predicted using mid-infrared (MIR) spectroscopy of a milk sample [3, 4]. Previous studies have reported genetic correlations between MIR predicted BUN (MBUN) and BUN that range from 0.90 [5] to 0.98 [6]. Hence, for the purpose of genetic analyses, MBUN and BUN can be considered as the same trait. Including MBUN in a reference population for genomic prediction can increase the prediction accuracy for BUN [6]. To further increase the accuracy of genomic prediction, genome-wide association studies (GWAS) could be used to select sequence variants associated with BUN. Including sequence variants among the prediction markers can increase the accuracy of genomic prediction [7–9]. Similar to genomic prediction, large datasets are required to increase the power of GWAS. While the power of a GWAS for BUN may be limited due to the difficulties in obtaining a sufficient number of samples, MBUN may provide an alternative to detect quantitative trait loci (QTL) associated with BUN. While several GWAS have been published for milk urea nitrogen (MUN) [10–12], a trait that is highly related to BUN [5, 13], to our knowledge, no GWAS has been published for either BUN or MBUN. Therefore, the objective of our study was to perform GWAS for BUN measured from blood samples and MBUN predicted from milk samples using spectroscopy, compare these two GWAS and detect QTL for both traits, and compare the detected QTL with previously reported QTL for MUN.

## Methods

Details of the BUN and MBUN phenotypes, and the genotypes used for this analysis have been previously described [5, 14]. We used one record per cow, with records from 2098 cows for BUN and from 18,120 cows for MBUN. The cows with BUN records were a subset of the cows with MBUN records. The majority of these records were from Holstein animals, but Jersey, Australian Red, Ayrshire and crossbred animals were also included in the dataset to maximise mapping power and precision [15]. While the number of Australian Red and Ayrshire animals was very small, they were included in the dataset because 987 of the crossbreds were part Australian Red and/or Ayrshire. Table 1 shows a breakdown of the number of records, their mean and standard deviation per breed for each trait. BUN was derived from blood samples, and MBUN from milk samples, according to the protocols described in Luke et al. [3] and Ho et al. [4].

**Table 1:** Number of records (N), mean and standard deviation (SD) per breed for blood urea nitrogen (BUN) and MIR predicted BUN (MBUN)

Breed	BUN			MBUN		
	N	Mean	SD	N	Mean	SD
Holstein	1569	5.6	2.1	12,660	4.9	1.9
Jersey	59	4.2	1.3	1857	5.8	1.6
Australian Red	2	5.3	0.5	95	6.9	2.2
Ayrshire	0	–	–	12	6.3	1.9
Crossbred	468	5.2	2.6	3496	4.8	2.1
All	2098	5.5	2.2	18,120	5.0	1.9

For all cows, high-density genotypes from the Bovine high-density (HD) Genotyping BeadChip and imputed whole-genome sequence data were available. The genotyping and imputation pipelines are described in van den Berg et al. [14]. Briefly, cows were genotyped with low- to medium-density single nucleotide polymorphism (SNP) panels. Raw genotypes were filtered based on the GenCall score, and then imputed to the BovineSNP50K BeadChip using a mixed Holstein and Jersey imputation reference population. Genotypes were subsequently further imputed to HD and finally to whole-genome sequence. The reference populations used for imputation to 50K, HD and sequence data included 14,722, 2700 and 4190 *Bos taurus* cattle, respectively. The latter corresponded to Run8 of the 1000 Bull Genomes Project [16, 17]. Missing genotypes in the reference whole-genome sequence data were imputed using the Beagle software v.4.1 [18], and only bi-allelic variants with an allele count of at least 3 and a Beagle R^2^ higher than 0.9 were retained. Imputation to 50K and HD was done using the Fimpute software v.3 [19], whereas imputation to sequence level was carried out with the Minimac4 software [20]. All variants were mapped to the ARS-UCD1.2 reference genome [21]. After filtering on minor allele frequency (MAF; ≥ 0.005) and Minimac imputation R^2^ (≥ 0.4), 15,625,438 SNPs were retained for subsequent GWAS.

HD genotypes (717,463 SNPs) were used to construct a genomic relationship matrix (GRM) and perform a principal component analysis (PCA) using the GCTA tool [22]. Scores for the first principal component (PC1) were included in the analyses to account for differences between the Holstein and Jersey breeds as described in van den Berg et al. [5]. PC1 was highly correlated (0.9997) with ADMIXTURE ancestry fractions [5]. Previous estimates [5] of fixed effects and covariates (the PC1, test month, herd-year-season, days in milk and age) were used to adjust phenotypes before performing the GWAS. GWAS was done using the GCTA tool [22], including the GRM (based on HD) for the following scenarios:

1. GWAS_BUN_ALL: GWAS for BUN, using all 2098 cows with BUN records;
2. GWAS_MBUN_ALL: GWAS for MBUN, using all 18,120 cows with MBUN records;
3. GWAS_MBUN_BUN: GWAS for MBUN, using only the records from the 2098 cows with BUN records;
4. GWAS_MBUN_2K, using the records from the 2000 randomly selected cows (repeated 5 times).

Because of the reduced dataset, GWAS_BUN_ALL, GWAS_MBUN_BUN and GWAS_MBUN_2K were carried out only for variants with a MAF ≥ 0.05. We considered that all the variants with a p-value ≤ 10–6 were significant, and calculated the false discovery rate (FDR) as $FDR=(nVariants \times {10}^{-6})/nSignificant$, where $nVariants$ is the total number of variants in the GWAS and $nSignificant$ is the number of variants in the GWAS with a p-value ≤ 10–6. QTL intervals were subsequently defined by grouping variants that were separated by less than 1 Mb in the same QTL interval. Genomic inflation factors of the GWAS were estimated using the “estlambda” function in the GenABEL R package [23]. To test the association between previously reported dairy cattle QTL [24, 25] and the QTL detected for BUN and MBUN, we repeated the GWAS for which QTL associated with BUN or MBUN were located in the same region as previously reported QTL including the previously reported QTL as covariates.

## Results and discussion

**Fig. 1:**
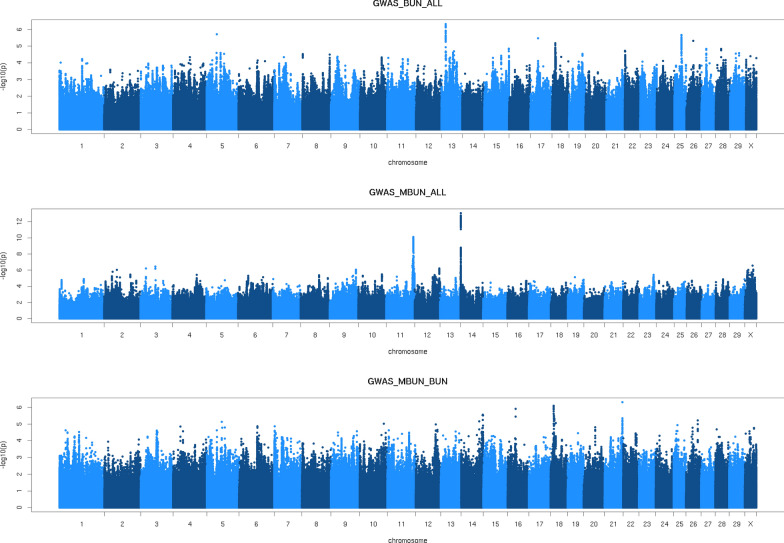
Manhattan plots of the GWAS for BUN and MBUN. *BUN* blood urea nitrogen, *MBUN* BUN predicted using mid-infrared spectroscopy, *GWAS_BUN_ALL* GWAS for BUN using all available BUN phenotypes (n = 2098), *GWAS_MBUN_ALL* GWAS for MBUN using all available MBUN phenotypes (n = 18,120), *GWAS_MBUN_BUN* GWAS for MBUN using only MBUN phenotypes of cows that also had BUN phenotypes (n = 2098)

In total, 640 and 5 significant variants were detected for MBUN and BUN, respectively, corresponding to an FDR of 0.02 and 2.27, respectively. Additional file 1: Figure S1 shows the Q-Q-plots of the observed and expected p-values in the GWAS for BUN and MBUN. Genomic inflation factors were equal 0.98 and 1.07 for BUN and MBUN, respectively. The much larger number of records for MBUN (18,120) than for BUN (2098) resulted in an increased power to detect QTL for MBUN compared to BUN (Fig. 1). Table 2 lists the QTL that were detected for each trait. Only one QTL on chromosome 13 was detected for BUN (15,837,206 bp, p_BUN_ = 4.8 × 10^–7^). The variant associated with this QTL was an intergenic variant, located between the *ENSBTAG00000048047* and *GATA binding protein 3* (*GATA3*) genes, and was not significant in the GWAS for MBUN (p_MBUN_ = 0.07). Given the extremely high FDR for BUN, this QTL is likely a false positive.

**Table 2:** Positions of potential QTL detected for BUN and MBUN

Chr	Pos (bp)	Trait	p_BUN_	p_MBUN_	Annotation	Genes	Start (bp)	End (bp)	N
2	47,485,307	MBUN	6.5 × 10^–1^	9.4 × 10^–7^	Intron	*EPC2*	47,485,307	47,485,307	1
3	18,190,277	MBUN	3.1 × 10^–2^	6.3 × 10^–7^	Upstream	*CRCT1*	18,190,277	18,190,277	1
3	55,238,179	MBUN	1.2 × 10^–3^	3.5 × 10^–7^	Intergenic	*PKN2-ENSBTAG00000051499*	55,238,179	55,257,795	33
9	97,711,991	MBUN	3.0 × 10^–2^	8.4 × 10^–7^	Intron	*PRKN*	97,711,991	97,711,991	1
11	99,897,676	MBUN	4.1 × 10^–1^	2.7 × 10^–7^	Intron	*ENSBTAG00000054738*	99,897,676	99,916,846	6
11	103,271,858	MBUN	7.7 × 10^–1^	7.7 × 10^–11^	Synonymous	*GLT6D1*	101,401,373	103,606,366	382
11	105,110,370	MBUN	8.0 × 10^–1^	6.8 × 10^–7^	Intron	*RXRA*	105,110,370	105,110,370	1
12	84,682,464	MBUN	2.8 × 10^–1^	6.0 × 10^–7^	Intergenic	*IRS2-RF00001*	84,682,431	84,699,022	3
13	15,837,206	BUN	4.8 × 10^–7^	6.6 × 10^–2^	Intergenic	*ENSBTAG00000048047-GATA3*	15,835,519	15,839,775	5
14	631,698	MBUN	1.5 × 10^–1^	8.6 × 10^–14^	Upstream	*BOP1*	512,818	1,278,273	209
X	31,752,695	MBUN	2.7 × 10^–2^	9.0 × 10^–7^	Intergenic	*ENSBTAG00000018311-IDS*	31,752,695	31,752,695	1
X	100,768,475	MBUN	4.2 × 10^–1^	2.7 × 10^–7^	Intergenic	*MAOA-PPP1R2C*	100,768,475	100,768,475	1
X	107,587,617	MBUN	3.1 × 10^–1^	8.7 × 10^–7^	Intergenic	*MAGEB16-ENSBTAG00000040406*	107,587,617	107,587,617	1

BUN, blood urea nitrogen; MBUN, BUN predicted using mid-infrared spectroscopy; chr, chromosome; pos, position of the most significant variant associated with the QTL; trait, trait for which the QTL is significant; bp, base pair according to the ARS-UCD1.2 annotation; p_BUN,_ p-value in the GWAS for BUN; p_MBUN_, p-value in the GWAS for MBUN; annotation, annotation of the most significant variant; gene, gene in which the most significant region was located or, if the most significant variant was intergenic, the genes between which the most significant variant was located, start, start of the QTL interval; end, end of the QTL interval; N, number of variants with p ≤ 10^–6^ in the QTL interval

We detected 12 QTL for MBUN, located on chromosomes 2, 3, 9, 11, 12, 14 and X. The most significant variant associated with MBUN was located at 631,698 bp on chromosome 14 (p = 8.6 × 10^–14^), upstream of the *BOP1 ribosomal biosis factor* (*BOP1*) gene. Several previous studies reported *BOP1* as a candidate gene associated with milk production traits [26–28]. The QTL interval on chromosome 14 encompassed the *diacylglycerol O-acyltransferase homolog 1* (*DGAT1*) gene, a well-known causal gene for milk production traits in dairy cattle [24]. After including the causal variant for the *DGAT1* QTL in the model as a fixed effect, none of the remaining variants on chromosome 14 were significant (see Additional file 2: Figure S2). Hence, *DGAT1* was in high LD with all variants within the peak and is likely the causal variant that underlies the QTL. The second most significant QTL was a synonymous variant in the *glycosyltransferase 6 domain containing 1* (*GLT6D1*) gene, located at 103,271,858 bp on chromosome 11. *GLT6D1* is associated with periodontitis in humans [29]. The QTL interval on chromosome 11 also included the *progestagen-associated endometrial protein* (*PAEP*) gene, a candidate gene for milk production traits in dairy cattle [25], and the *alpha 1–3-N-acetylgalactosaminyltransferase and alpha 1–3-galactosyltransferase* (*ABO*) gene, which has been reported as a candidate gene for protein yield in dairy cattle [30] and determines human blood type [31]. The most significant variant in the GWAS for MBUN was located in an intron of the *PAEP* gene at 103,262,933 bp. When including this variant as a fixed effect in the model, the large peak in the area disappeared (see Additional file 3: Figure S3), and none of the previously reported top variants for the QTL were significant anymore, which indicates that rather than the three QTL detected on chromosome 11, we detected only one QTL that is in high LD with *PAEP*. When the variant in *PAEP* was included as a fixed effect in the model, the only remaining significant variant was an intron in the *vav guanine nucleotide exchange factor 2* (*VAV2*) gene located at 104,765,599 bp with a p-value of 9.7 × 10^–7^. Ariyarathne et al. [10] reported a QTL for MUN in the same region on chromosome 11. MUN and MBUN had a genetic correlation of 0.77 in the dataset used in the current study [5], hence QTL for MBUN and MUN were expected to overlap. Both MBUN and MUN are derived using MIR spectroscopy data of a milk sample, which may contribute to the strong genetic correlation between MBUN and MUN and similarity in the GWAS results. In a GWAS for MUN using the same individuals as in our current analysis, QTL for MUN were detected that overlapped with QTL for MBUN on chromosomes 11, 14 and X [14]. On chromosome 11, the variant located at 103,271,858 bp was the most significant variant on this chromosome for both MBUN and MUN, with a p-value of 5.4 × 10^–16^ for MUN. The variants that were detected for MBUN at 631,698 bp on chromosome 14 and 107,587,617 bp on chromosome X have p-values of 1.2 × 10^–21^ and 1.1 × 10^–6^ for MUN, respectively (unpublished observations). MBUN had moderate genetic correlations with fat yield (0.28) and fat percentage (0.35) [5], which may explain the overlap between QTL detected for MBUN and the well-known milk production QTL on chromosomes 11 and 14 [24, 25]. However, given that the majority of the significant peaks identified in the GWAS for MBUN were associated with milk production, the GWAS results for any MIR-predicted trait may be heavily biased by milk characteristics. Several GWAS for MIR wave numbers have detected major QTL on chromosomes 3 [32], 11 [32–34] and 14 [32, 33, 35, 36] in the same regions where we detected QTL for MBUN.

The GWAS results for BUN and MBUN were very different, which was surprising given the genetic correlation of 0.90 between the two traits [5]. None of the QTL detected for MBUN were close to significance in the GWAS for BUN. Hence, it is possible that, in spite of the strong genetic correlation between MBUN and BUN, some of the variation in MBUN picked up in the GWAS is related to the variation of the MIR spectrum rather than to variation in BUN. Alternatively, the differences between the GWAS for MBUN and BUN may be due to the smaller subset of animals that had BUN records. To explore this, we repeated the GWAS for MBUN using only the animals with BUN phenotypes (GWAS_MBUN_BUN, Fig. 1). Using this dataset the peaks that we detected in the GWAS_MBUN_ALL analysis disappeared, which strongly suggests that the GWAS results were very sensitive to the set of individuals used. The dataset that we analysed contained multiple breeds, including crossbreds, hence the difference in GWAS results between subsets could also be due to differences in breed composition.

**Fig. 2:**
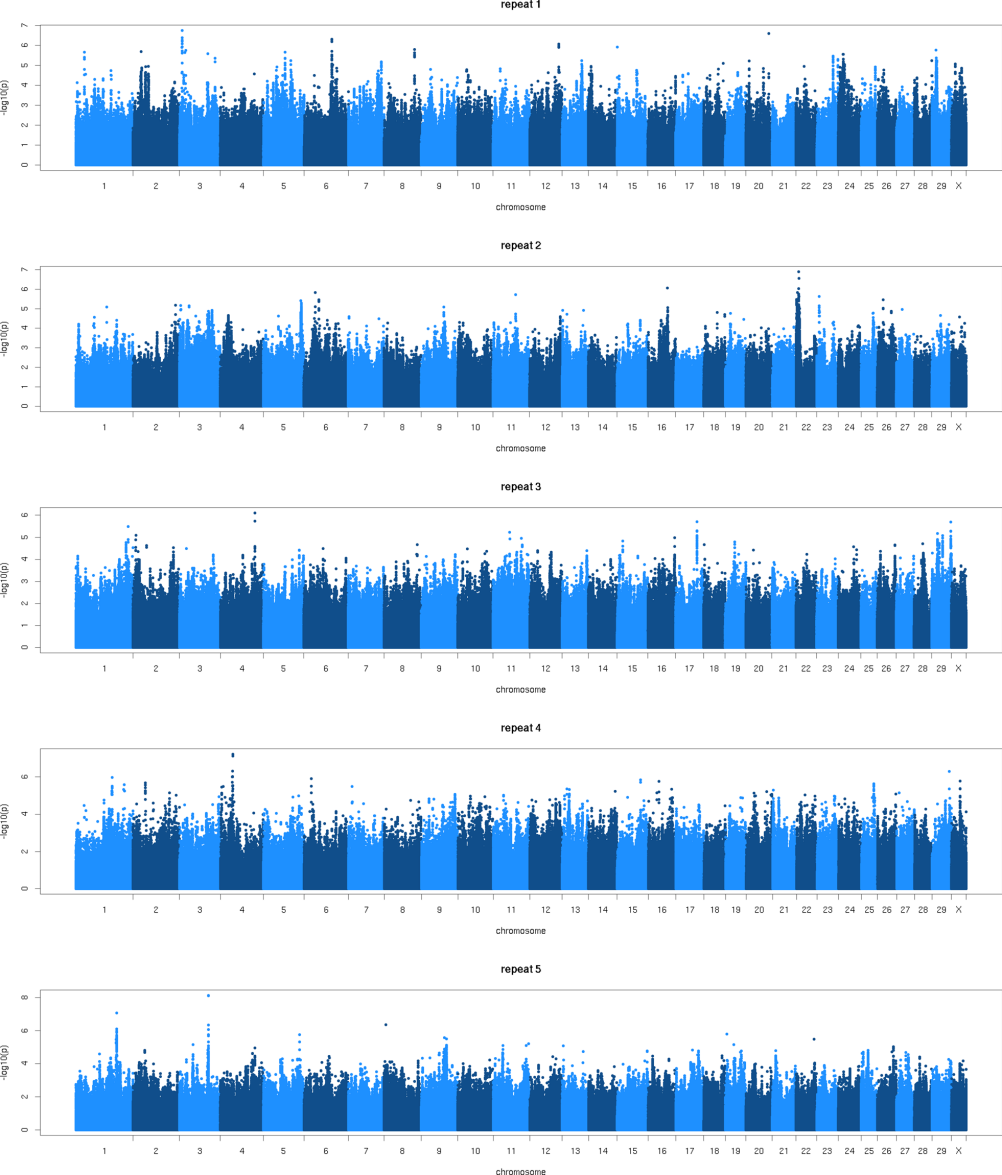
Manhattan plots of the GWAS for MBUN using different subsets of records. *MBUN* blood urea nitrogen (BUN) predicted using mid-infrared spectroscopy. The five repeats show GWAS carried out using MBUN phenotypes from 2000 randomly selected cows

To test if the differences between GWAS_MBUN_ALL and GWAS_MBUN_BUN were caused by (1) particular characteristics of the subset of animals with BUN records or (2) by the smaller sample size, we carried out a GWAS with records from 2000 randomly selected cows (GWAS_MBUN_2K), and as shown in Fig. 2, the results differed between datasets. None of the GWAS_MBUN_2K detected any of the larger QTL detected in the GWAS_MBUN_ALL. In each of the GWAS_MBUN_2K, small peaks with minimum p-values of approximately 10^–6^ to 10^–8^ were identified, but they were at different positions in each of the datasets. This implies that the GWAS results obtained by using small datasets (in this case, around 2000 cows) should be interpreted with caution, and larger datasets may be required to detect peaks that are less sensitive to the particular set of data analysed. A MIR predicted trait for which records can easily be generated for a large number of animals, such as MBUN, may provide an attractive alternative to perform a more powerful GWAS for hard-to-measure traits, such as BUN. Since there is a strong genetic correlation between MBUN and BUN, they can be considered as the same trait [5, 6], and hence the QTL detected for MBUN could be interpreted as QTL for BUN. This indicates the potential of using MIR equations from a breeding perspective. However, although Ho et al. [4] reported a comparable prediction accuracy from herd/animal independent validation and herd-year by herd-year validation, further research is required to develop prediction equations for MBUN that can be transfered across environments.

## Conclusions

The GWAS results for BUN and MBUN were very different, in spite of the strong genetic correlation between the two traits. We detected 12 QTL for MBUN, located on chromosomes 2, 3, 9, 11, 12, 14 and X. The QTL on chromosomes 11, 14 and X overlapped with previous QTL detected for milk production traits and/or MUN. We detected one QTL for BUN using a dataset of about 2000 cows that was located on chromosome 13. However, when we repeated the GWAS for MBUN on smaller (2000) subsets of the dataset i.e. so that their size was comparable to that for BUN, the GWAS results were very sensitive to the subset of records used. Hence, using approximately 2000 cow phenotypes as was done for the GWAS for BUN may not be sufficient for accurate QTL detection, and caution is warranted when interpreting GWAS results based on small datasets. Based on the strong genetic correlation between MBUN and BUN that was estimated in previous studies, MBUN may provide an attractive alternative to perform a more powerful GWAS to detect QTL for BUN.

## Supplementary Information

**Additional file 1: Figure S1.** Quantile–Quantile (Q–Q) plots of expected and observed p-values for the GWAS of blood urea nitrogen (BUN) and BUN predicted using mid-infrared spectroscopy (MBUN).

**Additional file 2: Figure S2.** GWAS for MBUN (blood urea nitrogen predicted using mid-infrared spectroscopy) on chromosome 14 including the causal variant for *DGAT1* as covariate.

**Additional file 3: Figure S3.** GWAS for MBUN (blood urea nitrogen predicted using mid-infrared spectroscopy) on chromosome 11 including an intron variant in *PAEP* as covariate.

## Data Availability

Data requests should be directed to DataGene Ltd (Melbourne, Australia) as custodians of data on Australian dairy cows. Research-related requests for access to the data may be accommodated on a case-by-case basis.
