# Supplementary figures and images for: Using mid-infrared spectroscopy to increase GWAS power to detect QTL associated with blood urea nitrogen
Source: Genet Sel Evol. 2022 Apr 18;54:27. doi: 10.1186/s12711-022-00719-5 (PMC9014603; doi:10.1186/s12711-022-00719-5)

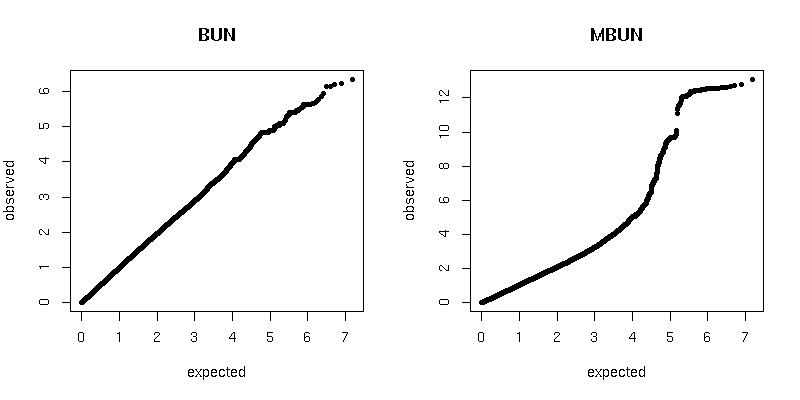

Supplement: Supplementary file 1 — Additional file 1: Figure S1. Quantile–Quantile (Q–Q) plots of expected and observed p-values for the GWAS of blood urea nitrogen (BUN) and BUN predicted using mid-infrared spectroscopy (MBUN). [file 12711_2022_719_MOESM1_ESM.png]

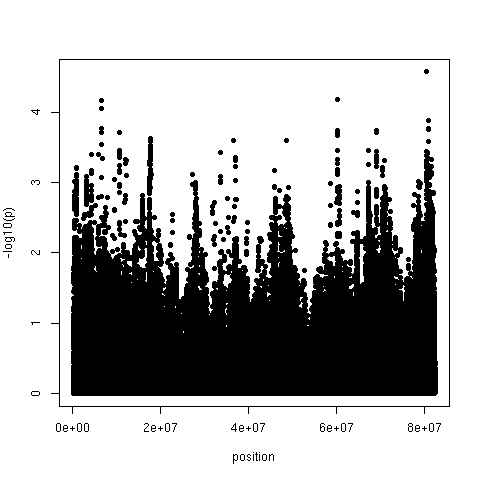

Supplement: Supplementary file 2 — Additional file 2: Figure S2. GWAS for MBUN (blood urea nitrogen predicted using mid-infrared spectroscopy) on chromosome 14 including the causal variant for DGAT1 as covariate. [file 12711_2022_719_MOESM2_ESM.png]

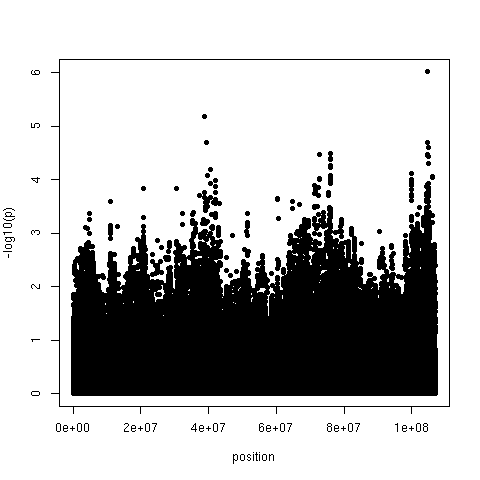

Supplement: Supplementary file 3 — Additional file 3: Figure S3. GWAS for MBUN (blood urea nitrogen predicted using mid-infrared spectroscopy) on chromosome 11 including an intron variant in PAEP as covariate. [file 12711_2022_719_MOESM3_ESM.png]
